# Study on the Improvement of Behavioral and Cognitive Dysfunction of Children with OSAHS by Vitamin D

**DOI:** 10.1155/2021/5536689

**Published:** 2021-05-07

**Authors:** Panpan Cui, Lei Ge, Jiansheng Li

**Affiliations:** ^1^Department of Otorhinolaryngology, People's Hospital of Rizhao, Jining Medical University, Shandong 276800, China; ^2^Department of Emergency, People's Hospital of Rizhao, Jining Medical University, Shandong 276800, China

## Abstract

**Objective:**

This study is aimed at exploring the role of vitamin D in the treatment of children with OSAHS by comparing the clinical symptoms, serum indicators, and behavioral changes of vitamin D intervention.

**Method:**

Healthy children who were examined physically in Rizhao People's Hospital were selected as the control group, and their sex, age, triglyceride, total cholesterol, high-density lipoprotein, low-density lipoprotein, serum 25-OHD levels, and Conners' parental scale were measured. In addition, children diagnosed as OSAHS in the otolaryngology department of Rizhao People's Hospital were selected as experimental subjects. Their body mass index, triglycerides, total cholesterol, high-density lipoprotein, low-density lipoprotein, serum 25-OHD levels, sleep apnea hypopnea index, minimum oxygen saturation, and Conners' parental scale were measured. The experimental subjects were treated with Rocaltrol intervention therapy (0.25 g/QD) for 4 weeks and reanalyzing their triglycerides, total cholesterol, high-density lipoprotein, low-density lipoprotein, serum 25-OHD levels, sleep apnea hypopnea index, minimum oxygen saturation, and Conners' parental scale.

**Result:**

OSAHS children commonly have the situation of obesity, dyslipidemia, and vitamin D deficiency. Behavioral and cognitive dysfunction is common in OSAHS children. There were no significant changes in body mass index, triglycerides, total cholesterol, high-density lipoprotein, low-density lipoprotein, sleep apnea, hypopnea index, and minimum oxygen saturation for OSAHS children after vitamin D treatment, but the serum 25-OHD level is significantly improved, and children with conduct problems, learning problems, and hyperactivity index decrease.

**Conclusion:**

(1) OSAHS children commonly have the situation of obesity, dyslipidemia, vitamin D deficiency, and behavioral and cognitive impairment. (2) Vitamin D supplementation has no therapeutic effect on obesity and dyslipidemia of OSAHS children, but has obvious protective and improving effects on neuron damage caused by hypoxia. Obstructive sleep apnea syndrome (OSAHS) is a common disease. Patients with OSAHS usually have many clinical features, such as obesity, metabolic syndrome, and cardiovascular disease. The decline of cognitive function and learning ability is one of the serious complications of OSAHS patients [1]. Vitamin D (VitD) deficiency is associated with many diseases. Recent studies have shown that the serum 25-OHD level of OSAHS patients is relatively insufficient and related to the disease severity. However, there are few reports on explaining whether vitamin D supplementation can alleviate the clinical symptoms and improve serum indicators and behavioral and cognitive dysfunction in children with OSAHS.

## 1. Data and Methods

### 1.1. General Information

In the OSAHS group, 30 children (including 22 males and 8 females) aged 7-13 years were selected from the otolaryngology department of Rizhao People's Hospital. In the control group, 20 children (including 16 males and 4 females) within the average age group of 6-12 years were selected from Rizhao People's Hospital, who were excluded from OSAHS through physical examination. The study was done after agreement from the local ethics committee and with the patients' informed consent. Rocaltrol , Roche Pharmaceutical Co Limited. Sleep Breathing Screening Instrument (DHR998), Dehaler Medical Technology Co Limited.

### 1.2. Research Methods

Gender, age, height, and weight of the control group were recorded. The fasting levels of triglycerides, total cholesterol, high-density lipoprotein, low-density lipoprotein, serum 25-OHD, and Conners' parental scale were measured in the control group.

Gender, age, height, and weight of the control group were recorded. The fasting levels of triglycerides, total cholesterol, high-density lipoprotein, low-density lipoprotein, serum 25-OHD, and Conners' parental scale were measured in the OSAHS group. Sleep monitoring was performed with a sleep breathing screening instrument (Dehaier Medical Technology Company,DHR998), and the sleep time was at least 7 h. By collecting PPG signals, blood oxygen, heart rate, sound of snoring, and posture signals, indexes like the hypopnea index (AHI) and lowest oxygen saturation (LSaO2) were automatically analyzed by computer. 7 mL peripheral venous blood was extracted on an empty stomach in the morning of the next day for sleep monitoring and then sent it to the central laboratory of the hospital immediately to detect triglycerides, total cholesterol, high-density lipoprotein, low-density lipoprotein, and serum 25-OHD levels. The experimental subjects were treated with Rocaltrol intervention therapy (0.25 *μ*g/QD) for 4 weeks. Reapplying sleep breathing screening instrument to collect AHI and LSaO2 and detecting the fasting levels of triglycerides, total cholesterol, high-density lipoprotein, low-density lipoprotein, and serum 25-OHD again the next morning.

### 1.3. Statistic Analysis

SPSS software was used to analyze these data. Data description was expressed as mean ± standard deviation (*x* ± *s*). *t*-test was used for pairwise comparisons, and the ANOVA test was used for multiple variables. *P* < 0.05 indicates statistical significance.

## 2. Results

### 2.1. Comparison between the Control Group and OSAHS Group

**Figure 1:**
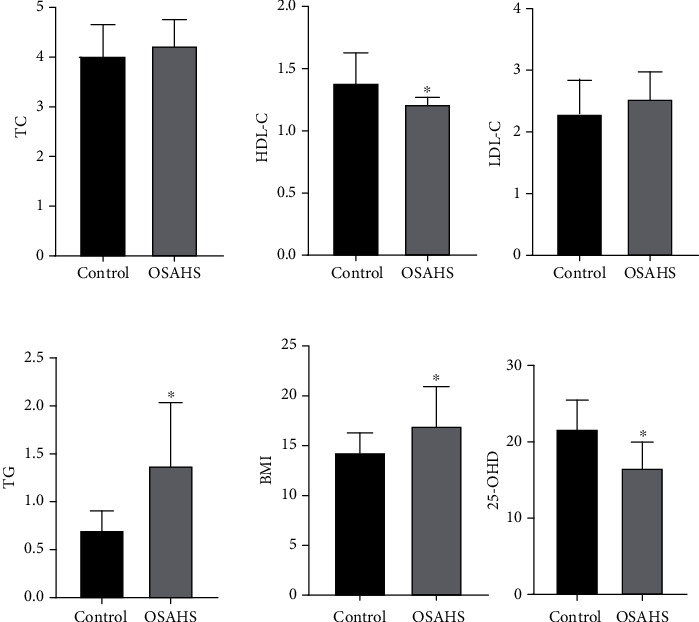
Comparison of serum indicators and BMI between the control group and OSAHS group. Indictors including triglyceride and body mass index of OSAHS group were obviously higher than those of the control group, while the level of serum 25-OHD and high-density lipoprotein of the OSAHS group was obviously lower than those of the control group. ^∗^*P* < 0.05 vs control. The results showed that OSAHS children commonly have the situation of obesity, dyslipidemia, and vitamin D deficiency.

**Figure 2:**
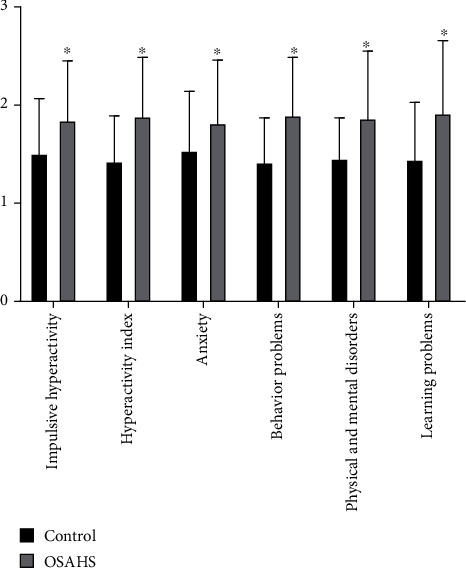
Comparison of Conners' parents between the control group and OSAHS group. Conners' parents of the OSAHS group were obviously higher than those of the control group. The differences were statistically significant. ^∗^*P* < 0.05 vs control. The results showed that OSAHS children commonly have behavioral and cognitive impairment.

Indictors including triglyceride, total cholesterol, low-density lipoprotein, and body mass index of the OSAHS group were clearly higher than those of the control group, while the level of serum 25-OHD and high-density lipoprotein of the OSAHS group was clearly lower than those of the control group. The differences were statistically significant (Figure 1). There are statistical significances for differences in behavior problems, learning problems, hyperactivity index, impulsive hyperactivity, anxiety, and physical and mental disorders among Conners' parents. The results showed that OSAHS children commonly have the situation of obesity, dyslipidemia, vitamin D deficiency, and behavioral and cognitive impairment (Figure 2).

### 2.2. The Correlation between the Severity of OSAHS and the Vitamin D Deficiency

**Figure 3:**
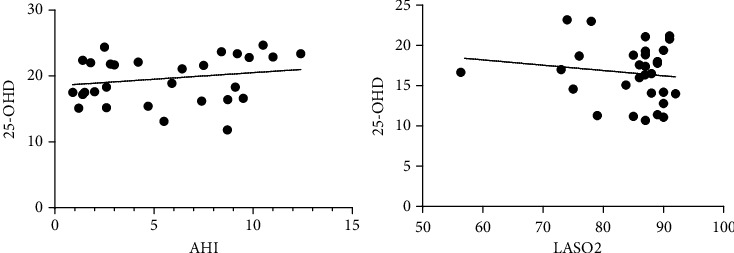
The correlation between the severity of OSAHS and the vitamin D deficiency. AHI and LSAO2 are important indicators to judge the severity of OSAHS. We counted the AHI, LSAO2, and 25-OHD of the OSAHS group and found no significant correlation between 25-OHD and AHI and LSAO2 (*R* < 0.5, *P* > 0.05).

AHI and LSAO2 are important indicators to judge the severity of OSAHS. We counted the AHI, LSAO2, and 25-OHD of the OSAHS group and found no significant correlation between 25-OHD and AHI and LSAO2 (*R* < 0.5, *P* > 0.05, Figure 3).

### 2.3. Comparison of Vitamin D Intervention Treatment before and after Treatment of the OSAHS Group

**Figure 4:**
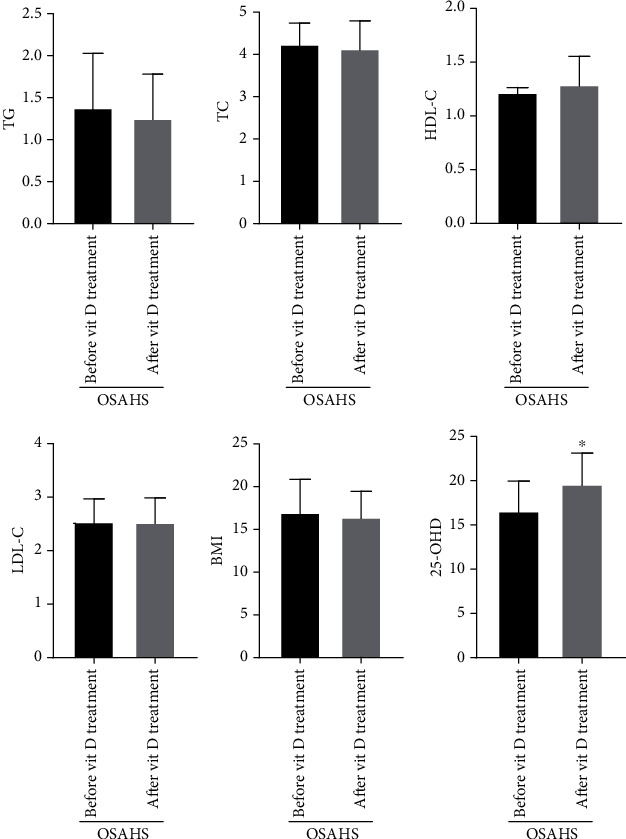
Comparison of vitamin D intervention treatment before and after treatment of the OSAHS group (serum indicators and BMI). The experimental subjects were treated with Rocaltrol intervention therapy (0.25 *μ*g/QD) for 4 weeks. The OSAHS group (after vitamin treatment) serum 25-OHD level is obviously higher than the OSAHS group (before vitamin treatment), whose difference has statistical significance. ^∗^*P* < 0.05 vs OSAHS group (after vitamin treatment). Comparison of children in the OSAHS group before and after vitamin D treatment has no significant changes in triglyceride, total cholesterol, low-density lipoprotein, high-density lipoprotein, and body mass index, whose difference has no statistical significance.

**Figure 5:**
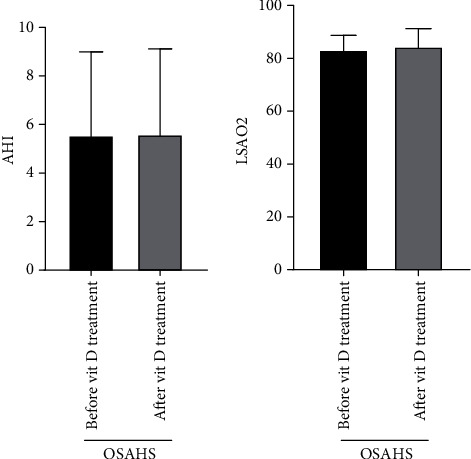
Comparison of vitamin D intervention treatment before and after treatment of the OSAHS group (AHI and LSAO2). The experimental subjects were treated with Rocaltrol intervention therapy (0.25 *μ*g/QD) for 4 weeks. Comparison of children in the OSAHS group before and after vitamin D treatment has no significant changes in AHI and LSAO2, whose difference has no statistical significance (*P* > 0.05).

**Figure 6:**
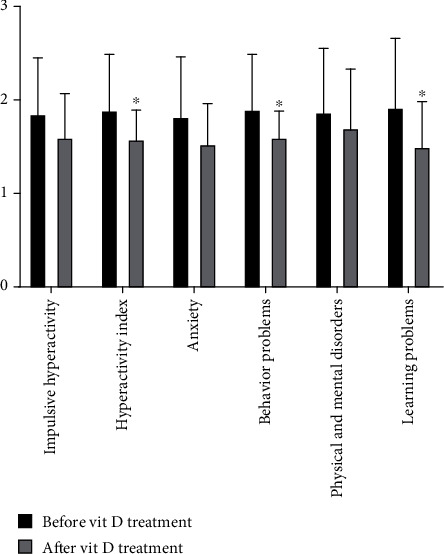
Comparison of vitamin D intervention treatment before and after treatment of OSAHS (Conners' parents). The experimental subjects were treated with Rocaltrol intervention therapy (0.25 *μ*g/QD) for 4 weeks. During the comparison of children in the OSAHS group before and after vitamin D treatment, there are statistical significance for differences in behavior problems, learning problems, and hyperactivity index among Conners' parents. ^∗^*P* < 0.05 vs OSAHS group (after vitamin treatment). There was no statistical significance for difference in impulsive hyperactivity, anxiety, and physical and mental disorders.

The OSAHS group (after vitamin treatment) serum 25-OHD level is clearly higher than the OSAHS group (before vitamin treatment), whose difference has statistical significance. Comparisons of children in the OSAHS group before and after vitamin D treatment had no significant changes in AHI, LSAO2, triglyceride, total cholesterol, low-density lipoprotein, high-density lipoprotein, and body mass index, whose difference has no statistical significance (*P* > 0.05, Figures 4 and 5). During the comparison of children in the OSAHS group before and after vitamin D treatment, there are statistical significance for differences in behavior problems, learning problems, and hyperactivity index among Conners' parents. There was no statistical significance for the difference in impulsive hyperactivity, anxiety, and physical and mental disorders (Figure 6). The result shows that vitamin D deficiency is common in OSAHS children, and after treatment with vitamin D, the situation has been significantly improved. Vitamin D has no therapeutic effect on obesity and dyslipidemia in OSAHS children. In addition, behavioral and cognitive dysfunction is common in OSAHS children, and the behavioral and cognitive dysfunction of OSAHS children is significantly improved after treatment with vitamin D intervention.

## 3. Discussion

Previous studies have shown that vitamin D deficiency is common in OSAHS patients. Archantogeorgis studied 88 patients with OSAHS, 55 of whom had vitamin D deficiency [2]. Kerley investigated 106 adult OSAHS patients in Dublin, Ireland, and found that 98% of OSAHS patients had 25-OHD deficiency (<75 nmol/L), including 72% of OSAHS patients with 25-OHD deficiency (<50 nmol/L) [3]. Vitamin D receptor widely exists in muscle. It can promote the proliferation and differentiation of muscle cells by regulating a variety of transcription factors, especially the transformation of type II muscle fiber tissue. At the same time, Vitamin D can promote the transfer of Ca^+^ to the endoplasmic reticulum, to regulate the contraction of muscle fibers. Vitamin D deficiency in patients may lead to abnormal muscle contraction, which may be also one reason for OSAHS. In addition, enzymes related to vitamin D metabolism and vitamin D receptors widely exist in various immune cells, including antigen-presenting cells, T cells, B cells, and monocytes. They regulate the related immune function by regulating related cytokines. In addition, vitamin D can inhibit the secretion of IL-2, TNF-*α*, and IFN-*γ* by Th-1 and promote the secretion of IL-4, IL-3, IL-5, and IL-10 by Th2, to inhibit the occurrence of chronic inflammation, which plays an important role in the occurrence and development of OSAHS. In addition, OSAHS patients have declining sleep quality, which may lead to daytime sleepiness or obesity, reduced outdoor activities, resulting in insufficient vitamin D synthesis, and then vitamin D deficiency. In this study, through the research on the serum 25-OHD level in normal children and OSAHS children, the prevalence of vitamin D deficiency in OSAHS children will be further explored, which is also consistent with previous studies.

Polysomnography is the “gold standard” for the diagnosis of OSAHS. Respiratory disorder index and the lowest blood oxygen saturation in the monitoring report are important indicators for the immediate diagnosis of OSAHS, as well as important aspects for judging the severity of OSAHS. As for the relationship between OSAHS severity and vitamin D, a lot of previous researches have been done, but these results are often contradictory. By studying 139 OSAHS patients, Archontogeorgis found that the serum 25-OHD level of these patients was significantly lower than that of healthy people which was negatively correlated with AHI and blood oxygen saturation, indicating that the VitD level of OSAHS patients was low, and the severity of OSAS was also related to the VitD level [4]. Bozkurt et al. found that the serum vitamin D level of patients decreased slightly with the severity of OSAHS, but there was no statistically significant difference [5]. Through multivariate logistic regression analysis, Sharma thought that 25-OHD was significantly correlated with OSAHS, but he did not indicate the correlation between 25-OHD and AHI [6]. This research used a double-blind, control group and experimental group to study that vitamin D deficiency was prevalent in OSAHS children. However, appropriate supplementation of vitamin D to OSAHS children did not significantly improve the condition of OSAHS.

OSAHS is generally characterized by obesity and dyslipidemia. Under the influence of repeated hypoxemia, the secretion of growth hormone by the hypothalamus may be reduced, resulting in lipid metabolism disorder and forming a vicious circle. Savastano indicated that the reduction of vitamin D production in obese people due to sedentary lifestyle and less outdoor activities, as well as the large volume dilution of vitamin D in fat, serum, liver, and muscle, is all important reasons of serum vitamin D deficiency and insufficient.[7].

Wamberg's study showed that obese people, who have larger surface areas and eat more, should have increased intake and synthesis of vitamin D. However, serum 25-OHD levels of obese people were significantly decreased, which may be related to the fact that obese people have less outdoor activities. Vitamin D is stored in large quantities in the adipose tissue, where the enzymes responsible for activating vitamin D are low in expression and tend to be catabolic. These are the causes for the low serum 25-OHD level in obese people [8]. Results of the current study are inconsistent with the impact of vitamin D deficiency on obesity. Kang et al. interfered with rats through vitamin D and found that supplementing appropriate vitamin D significantly reduced the weight of rats, which may be because vitamin D negatively regulates the expression of various fat-producing genes in the adipose tissue and liver, including fatty acid synthase (FAS), stearyl coa desaturase 1(SCD1), and acetyl-coa carboxylase 1(ACC1) [9]. Other findings do not support vitamin D deficiency as a cause of obesity. Through supplementing nonobese subjects with calcitriol and the serum 1,25 (OH) D3 concentration increased, Boon found that enzyme and gene expression of lipid metabolism-related proteins had no apparent effect [10]. This study shows that obesity and dyslipidemia are generally prevalent in OSAHS children. Moreover, appropriate vitamin D supplementation in OSAHS children showed no effect on BMI and blood lipid levels.

OSAHS children often show more behavioral and psychological problems due to long-term sleep, hypoxia, and sleep structure disorder. There are many tests to measure children's behavior. We choose the Conners' parenting scale as a measure tool of neurobehavioral outcomes, because it is an effective, short, comprehensive, age, and gender-specific measurement method. After Conners' parents' questionnaire is introduced into clinical use in China, many times of domestic standardization, it has good reliability and validity. Conners' parents used six factors of the questionnaire to summarize children's common behavior problems, which is the most widely used scale to evaluate children's behavior problems. Long-term chronic intermittent hypoxia can lead to brain damage in OSAHS children, and the hippocampus is recognized as a brain area closely related to learning and memory, and it is very sensitive to hypoxia, especially CA1 area; so, chronic intermittent hypoxia leads to hypoxia damage in the hippocampus, leading to cognitive dysfunction. After the application of CPAP treatment, Rosenzweig found that the neurocognitive structure, daytime sleepiness, and verbal episodic memory of OSAHS patients are improved [11]. The research of Yu found that OSAHS children had poorer intelligence, memory, attention, executive function, and language ability than the healthy babies. After surgical treatment, the attention, executive function/language ability, and memory of OSAHS children are significantly improved [12]. The research of Mir found that children with sleep disorders have impaired intelligence and behavior, expressing that the scores of words, reasoning ability, and intelligence development are significantly lower than those of normal children. After treatment, the cognitive function is partially improved [13]. This study found that vitamin D deficiency and abnormal Conners' parental scale score are common in OSAHS children. We found that the behavior problems, learning problems, and hyperactivity index of OSAHS children are significantly improved by appropriate vitamin D supplementation. We found that this phenomenon is consistent with the changes of some children's behavior problems caused by chronic hypoxia after treatment.

In conclusion, this indicates that vitamin D has no obvious improvement effect on the severity of OSAHS, but it has an obvious protection and improvement effect on neurons damaged by hypoxia. However, the mechanism of VitD deficiency and neurologic impairment induced by OSAHS needs further exploration and so is the mechanism of protecting hippocampal neurons from VitD.

## Data Availability

The clinical data used to support the findings of this study are restricted by the hospital in order to protect patient privacy. Data are available from Lei Ge for researchers who meet the criteria for access to confidential data, e-mail: 178603499@qq.com.
